# Endophytic Bacteria and Essential Oil from *Origanum vulgare* ssp. *vulgare* Share Some VOCs with an Antibacterial Activity

**DOI:** 10.3390/microorganisms10071424

**Published:** 2022-07-14

**Authors:** Giulia Polito, Giulia Semenzato, Sara Del Duca, Lara Mitia Castronovo, Alberto Vassallo, Sofia Chioccioli, Duccio Borsetti, Vittoria Calabretta, Anna Maria Puglia, Renato Fani, Antonio Palumbo Piccionello

**Affiliations:** 1 Department of Biological, Chemical and Pharmaceutical Sciences and Technologies (STEBICEF), University of Palermo, Viale delle Scienze Ed 17, 90128 Palermo, Italy; giulia.polito@unipa.it (G.P.); a.maria.puglia@unipa.it (A.M.P.); 2 Department of Biology, University of Florence, Via Madonna del Piano 6, Sesto Fiorentino, 50019 Florence, Italy; giulia.semenzato@unifi.it (G.S.); sara.delduca@unifi.it (S.D.D.); l.castronovo@student.unisi.it (L.M.C.); alberto.vassallo@unicam.it (A.V.); sofia.chioccioli@unifi.it (S.C.); duccio.borsetti@stud.unifi.it (D.B.); vittoria.calabretta@stud.unifi.it (V.C.); renato.fani@unifi.it (R.F.)

**Keywords:** volatile organic compounds, endophytes, antibacterial, medicinal plants

## Abstract

Medicinal aromatic plants’ essential oils (EOs) are mixtures of volatile compounds showing antimicrobial activity, which could be exploited to face the emerging problem of multi-drug resistance. Their chemical composition can depend on the interactions between the plant and its endophytic microbiota, which is known to synthesize volatile organic compounds (VOCs). However, it is still not clear whether those volatile metabolites can contribute to the composition of the aroma profile of plants’ EOs. The aims of this study were to characterize medicinal plant *O. vulgare* ssp. *vulgare* bacterial endophyte VOCs, evaluating their ability to antagonize the growth of opportunistic human pathogens belonging to the *Burkholderia cepacia* complex (Bcc) and compare them with *O. vulgare* EO composition. Many of the tested endophytic strains showed (i) a bactericidal and/or bacteriostatic activity against most of Bcc strains and (ii) the production of VOCs with widely recognized antimicrobial properties, such as dimethyl disulfide, dimethyl trisulfide, and monoterpenes. Moreover, these monoterpenes were also detected in the EOs extracted from the same *O. vulgare* plants from which endophytes were isolated. Obtained results suggest that endophytes could also play a role in the antibacterial properties of *O. vulgare* ssp. *vulgare* and, potentially, in determining its aromatic composition.

## 1. Introduction

Many microorganisms from different natural environments, especially bacteria, produce a wide range of info-chemicals, most of which are volatile compounds (VOCs) [1], a class of heterogeneous natural molecules formed via primary and secondary metabolic pathways [2]. Generally, VOCs are odorous compounds with low molecular mass (<300 Da), high vapor pressure, low boiling point, and a lipophilic moiety [3]. These compounds can belong to different chemical classes such as alcohols, esters, hydrocarbons, terpenes, ketones, sulphur-containing compounds, and carboxylic acids [2].

The microbial species and the substrate composition are the most important factors that can affect the production of these compounds [4]. The substrate composition has a great influence on both qualitative and quantitative production of volatile metabolites [2]. Moreover, moisture and temperature act on the emission of VOCs and they may affect the production of certain compounds and extend the time of maximum production. Other environmental factors such as pH of the substrate, light, and levels of CO_2_ or O_2_ probably also influence the VOC pattern [5].

The function of VOCs has not been clearly defined yet. Several studies have shown that bacterial volatile compounds constitute an important regulatory factor in determining intra- or interspecific communication and in chemical defence against other microorganisms. The production of VOCs by soil microorganisms is likely to have an important influence on atmospheric chemistry, soil processes, and biotic interactions in soil [6].

VOCs are suitable for both short- and long-distance signalling because of their capacity to diffuse through gas and water-filled pores within the heterogeneous soil matrix [3,7,8].

Under competitive soil conditions, due to the presence of other competing organisms, VOCs are important for antibiosis and signalling for symbiotic interactions [9].

The ability of these compounds to suppress pathogens and signal to plants proves their potential to be exploited as alternatives to chemical fertilizers and pesticides, which could provide a more sustainable solution as well as have negligible hazardous effects on animals and the environment [10]. Moreover, recent studies have established that VOCs are able to influence the growth of other bacteria, in a sort of chemical at-a-distance cross-talk [11,12,13].

These compounds are also important in the marine environment due to their ability to diffuse through aqueous solutions [2]. It has been previously demonstrated that Antarctic bacteria belonging to different genera/species are able to synthesize VOCs of different chemical classes, including sulphur compounds [2,14,15,16]. Interestingly, VOCs synthesised by several Antarctic bacteria specifically inhibit the growth on solid media of strains belonging to the *Burkholderia cepacia* complex (Bcc), a group of opportunistic human pathogens, most of which belong to the Multi-Drug Resistance (MDR) class [17]. In particular, bacteria belonging to the Bcc are pathogens in cystic fibrosis (CF) patients and are resistant to a plethora of antibiotics. A preliminary analysis performed on a set of Antarctic bacteria belonging to the genera *Pseudoalteromonas*, *Psychrobacter*, and *Shewanella* has revealed that the synthesis of VOCs is constitutive, in that it is not induced by the presence of target strains [15]. Given the fact that Antarctic bacteria previously analysed by Papaleo et al. [14] did not affect the growth of other classical bacterial human pathogens at all, it has been strongly suggested that the Bcc strains possess a metabolic trait that is sensitive to one or more VOCs produced by Antarctic bacteria, so these compounds might represent new antimicrobial compounds able to face CF patient infections [17].

The finding that secondary metabolites synthesized by microorganisms can inhibit the growth of pathogenic bacteria has been confirmed by recent data regarding strains isolated from different compartments of medicinal plants [18,19,20]. The use of medicinal and aromatic plants has a very ancient history in the prevention and treatment of mental and physical illnesses, also due to the production of essential oils (EOs). Synthesized as secondary metabolites, EOs appear as mixtures of volatile compounds. They are mainly involved in the protection of their host from phytopathogens and herbivores, and in the attraction of insect pollinators [21]. The antimicrobial activity of various EOs has been demonstrated, and nowadays, more efforts need to be addressed to the discovery of alternative bioactive molecules able to inhibit MDR human pathogens. In this scenario, major constituents of aromatic plant EOs can potentially be employed as antibacterial and antifungal agents [22].

To better exploit the pharmacological potential of medicinal plants and their EOs, it is essential to consider them as a dynamic and complex system; beside abiotic factors, their chemical composition also depends on the interactions between the plant and its endophytic microbiota [23]. Bacterial endophytes are microorganisms that inhabit the internal tissues of a plant without causing any sign of infection. The relationship between medicinal aromatic plants and their microbiota seems to rely on the production of secondary metabolites by both the plant and the bacteria: the plant selects the microorganisms that best adapt to its inner compartments, while the endophytes can modify these microenvironments influencing the plant secondary metabolism [23,24,25]. The endophytic community might also be directly responsible for the production of VOCs [26], but it is still not clear whether those volatile metabolites can actually contribute to the composition of the aroma profile of aromatic plants’ EOs.

Considering their potential biotechnological applications as aroma producers or drug sources, endophytic bacterial strains have been isolated from *Origanum vulgare* ssp. *vulgare* aerial parts, as described in Castronovo et al. [27]. The endophytes were tested for their antibacterial activity against Bcc strains via cross-streaking tests; eight of them showed a relevant growth inhibitory ability, probably due to the production of antibacterial bioactive compounds that diffused through the agar culture medium [27]. Little is known about the chemical nature of the produced metabolites, and one may ask if the antibacterial effect can also be attributed to the production of bacterial VOCs.

In the present study, the eight endophytic strains were characterized by means of both volatile-mediated inhibitory activity assay and solid phase micro extraction (SPME) coupled with gas chromatography–mass spectrometry (GC–MS) in order to investigate their ability to produce VOCs able to antagonize the growth of opportunistic human pathogenic Bcc strains. Moreover, the obtained VOC profiles were compared with the aroma profile of the EO extracted from the same *O. vulgare* plants from which the endophytes were isolated [27], in order to check whether any bacterial compound might contribute to the EO composition.

## 2. Results

### 2.1. VOCs Synthesized by O. vulgare Endophytes Possess Antibacterial Activity

**Figure 1:**
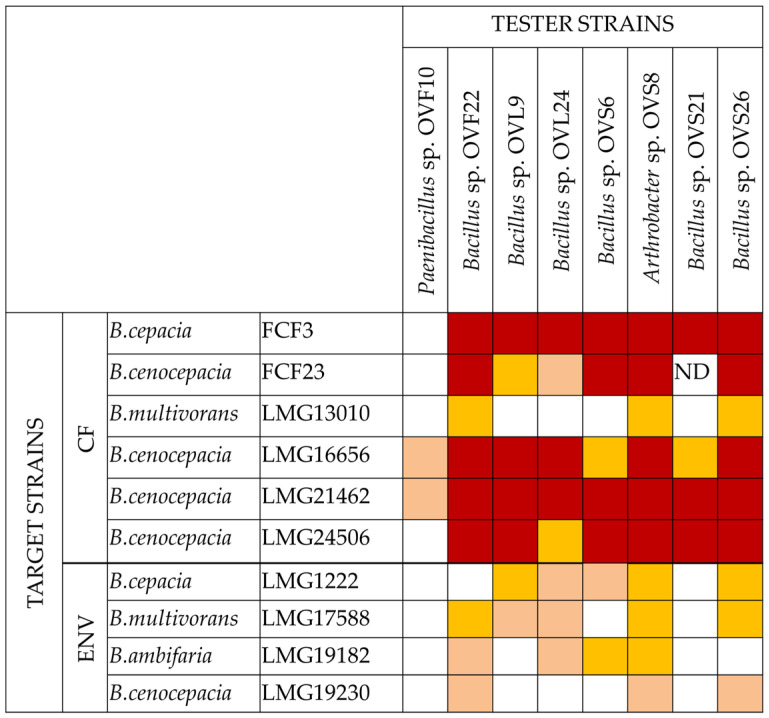
Antibacterial activity of *O. vulgare* ssp. *vulgare* associated endophytes against Bcc strains. The experiments were repeated twice. The inhibition values reflect three different inhibition levels, that is, complete (red), strong (orange), weak (salmon), and absence of inhibition (white). ND (not detected) refers to results that were not obtained. The origin of the target strains is referred to as CF (cystic fibrosis patients) and ENV (environmental).

*O. vulgare* ssp. *vulgare* endophytic strains were tested for their VOC production potential through the cross-streaking method, using Petri dishes with a septum physically separating the culture plate into two compartments, as described in Materials and Methods. Ten Bcc strains (either isolated from the environment or from cystic fibrosis patients) belonging to four species, *B. cepacia*, *B. cenocepacia*, *B. multivorans*, and *B. ambifaria*, were chosen as targets for the inhibition test. The eight tester strains were chosen from a larger panel of bacterial endophytes since they showed a high antagonistic activity against Bcc and/or other human pathogens (Coagulase-Negative Staphylococci, *Staphylococcus aureus,* and *Klebsiella pneumoniae*), when tested on Petri dishes without the separating septum [27]. The data obtained are shown in Figure 1, whose analysis revealed that all tester strains exhibited a complete or strong growth inhibiting capacity against target strains of human origin, except for OVF10. Finally, target strains LMG13010 and LMG19230, belonging to *B. multivorans* and *B. cenocepacia* species, respectively, showed the highest resistance profile, being inhibited by OVF22, OVS8, and OVS26 only.

### 2.2. Quantitative Analysis of VOCs Antibacterial Activity

**Figure 2:**
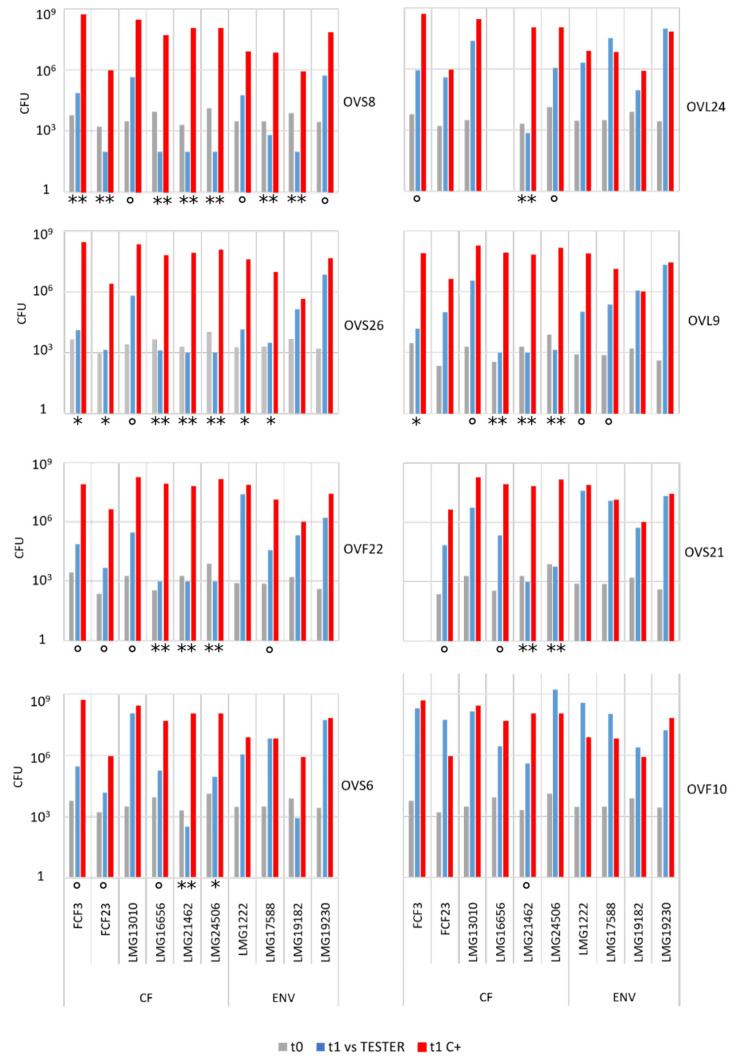
Schematic representation of the number of colony-forming units (CFU) of each target strain calculated at the beginning (t_0_, grey bar) and at the end (t_1_) of the cross-streaking experiments in the absence (red bar) or in the presence (blue bar) of the endophytic tester strains. The antibacterial activity exerted by the endophytic VOCs was classified as follows: (**) bactericidal, if the viable titer of target strain in the presence of the tester was lower than 0.015%; (*) bacteriostatic, when the viable titer was between 0.015% and 0.05%; (°) able to reduce the growth of the target, for values between 0.05% and 2%. The experiments were repeated twice.

**Figure 3:**
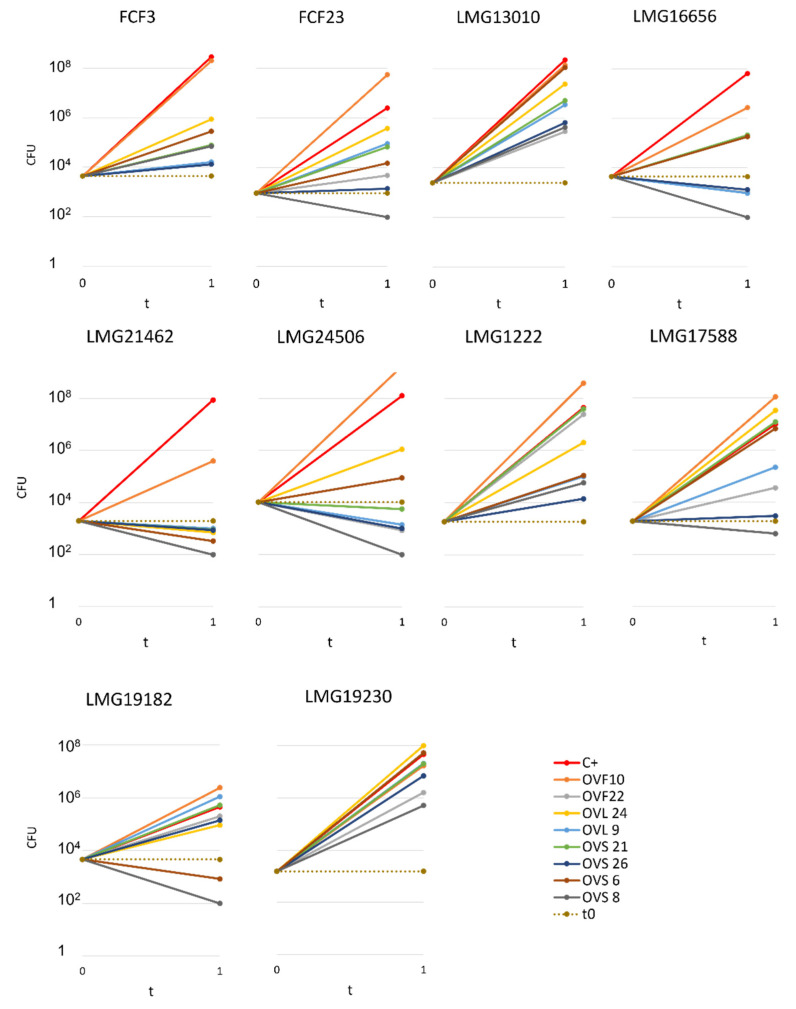
*O. vulgare* ssp. *vulgare* endophytes’ VOC effect on target strains’ growth.

Endophyte antibacterial activity was quantified through the cross-streaking method in order to permit the determination of the number of viable cells of each target strain at the beginning (i.e., soon after their streaking on plates, t_0_) and at the end of the experiment (i.e., after 48 h incubation in the presence of the tester strain, t_1_). These experiments were performed according to the protocol detailed in Materials and Methods. In principle, five different scenarios can be depicted: (i) the presence of the VOCs produced by the tester strain do not interfere with the growth of the target strain (s); (ii) the tester strain might reduce the growth rate of the target strains; (iii) the tester strain synthesizes VOCs able to completely interfere with the growth of the target cells (i.e., they exhibit a bacteriostatic activity); (iv) the VOCs synthesized by the tester strain might kill the target cells (i.e., they possess a bactericidal ability); (v) some VOCs might have a bacterial growth promoting ability that cannot a priori be excluded. The whole body of data obtained is reported in Supplementary Table S1 and is schematically represented in Figure 2 and Figure 3 and in Table 1.

Overall, the quantitative analyses revealed the following:

(i) All the tester strains were able to inhibit the growth of at least one of the target strains.
(ii) The VOCs synthesized by the eight endophytes exhibited a different ability to interfere with the growth of target strains.
(iii) The most active endophytic strain was *Arthrobacter* sp. OVS8, whose VOCs were able to strongly antagonize the growth of all the Bcc strains, showing a bactericidal activity against seven out of ten Bcc strains (see Figure 2 and Figure 3), and a growth reducing activity on the remaining three targets. Overall, the mean number of viable cells of target strains was 0.17% in the presence of strain OVS8 with respect to the control plates (i.e., in the absence of the tester strain).
(iv) On the other site, *Paenibacillus* sp. OVF10 exhibited the weakest antibacterial potential, being able to antagonize only the growth of LMG16656 and LMG21462 strains. These two targets, together with LMG24506, appeared to be the most sensitive to the VOCs produced by all the endophytes. It is also quite interesting that OVF10 is the only tester strain able to stimulate the growth of four targets (FCF23, LMG24506, LMG1222, and LMG17588).
(v) The remaining six tester strains, all belonging to the *Bacillus* genus, showed a different inhibitory effect on Bcc strains. All *Bacillus* strains induced a bactericidal effect against at least one of the target strains, with OVS26 and OVF22 exerting the strongest antibacterial activity.
(vi) The clinical Bcc strains isolated from CF patients were more sensitive to the endophytic VOCs than the environmental ones.
(vii) The two *B. multivorans* strains (one of clinical origin and the other one isolated from the environment) were less sensitive to the endophytic VOCs than the other eight Bcc strains belonging to the *B. cepacia*, *B. cenocepacia*, and *B. ambifaria* species.

### 2.3. SPME-GC/MS Analysis of VOCs

For each endophytic strain, the chemical profiles of identified VOCs detected by SPME-GC/MS are listed in Table 2. Results are expressed as a percentage of the VOC by dividing the area of each peak by the total area of the chromatogram peaks for each endophytic strain.

Solid phase microextraction (SPME) was chosen to analyse volatile compounds as it appears to be a technique that fits most of the requisites for sample preparation steps due to its simplicity, no solvent use, little sample manipulation, multiple sampling, high sensitivity, lower analysis time interval, and ease of automation [28].

A total of 34 distinct metabolites were detected from VOCs of *O. vulgare* ssp.* vulgare* strains. The major identified compounds included the following structurally distinct classes: sulfides, organic acids, ethers, terpenes, aromatic and aliphatic hydrocarbons, alcohols, pyrazines, esters, ketones, and aldehydes.

## 3. Discussion

In this work, we have determined the volatile organic compound (VOC) profile emitted by eight Gram-positive endophytic strains isolated from the medicinal plant *Origanum vulgare* ssp. *vulgare*, in an attempt to correlate them with the antibacterial activity exhibited by each endophyte against ten representatives of the *Burkholderia cepacia* complex (Bcc), which includes important human opportunistic pathogens able to infect immunocompromised patients, such as those affected by cystic fibrosis. In addition to this, another aim of this work was to compare the VOCs profile of each endophyte with the essential oil (EO) extracted from the same *O. vulgare* plants from which the endophytes were isolated (for a detailed description of the isolation methodology, see Castronovo et al. [27]) in order to check whether one (or more) compound/s was/were shared between the EO and the VOCs emitted by the endophytic bacteria.

The whole body of data obtained revealed that, even though isolated from the same plant, and in some cases from the same compartment of the same plant, the eight endophytes exhibited different anti-Bcc activities, with one of them (i.e., *Arthrobacter* sp. OVS8, isolated from the stem of *O. vulgare*) showing the highest and very strong bactericidal activity against most of the Bcc strains, and a bacteriostatic or a reducing growth ability on the other ones. On the other hand, *Paenibacillus* strain OVF10 did not exert any antagonistic activity towards the Bcc bacteria. The two strains had very different VOC profiles, suggesting that their composition and/or the relative concentration of their compounds might represent key players for the anti-Bcc activity.

The other six endophytic strains, all belonging to the genus *Bacillus*, did not exert the same anti-Bcc activity, even though all of them showed strong bactericidal activity towards the *B. cenocepacia* clinical isolate LMG21462, representing the most sensitive target strain to the tester VOCs. In spite of this common anti-LMG21462 bactericidal activity, the six *Bacillus* strains differentially inhibited the growth of the other target strains. Hence, the whole body of data obtained in this work revealed that all the five scenarios that were predicted, i.e., (i) no activity vs Bcc strains, (ii) reducing growth, (iii) bacteriostatic activity, (iv) bactericidal activity, and (v) promoting growth, actually occurred.

In order to check whether the different anti-Bcc activities might be related to a different VOC profile and/or to different concentrations of the compounds, the VOCs produced by the eight endophytes were determined. Based on the relative peak area, dimethyl disulfide was the most abundant volatile compound for all the endophytic strains, followed by 2-methyl-2-butene. Among sulfides, dimethyl trisulfide was also detected, which is a metabolite common to all strains. It was demonstrated that dimethyl disulfide and dimethyl trisulfide cause significant growth inhibition on different pathogens such as *Rhizoctonia solani* and *Pythium ultimum* and many Gram-negative and Gram-positive bacteria [29,30]. Therefore, the highest percentage of dimethyl disulfide (84.55%) detected in *Arthrobacter* sp. OVS8 is consistent with the observation that this endophytic strain is the most active one. This finding is also in agreement with previous data on VOCs synthesized by Antarctic bacteria exhibiting strong antibacterial activity against Bcc strains [14,15]. Indeed, some of the compounds belonging to the VOC profile of the endophytes tested in this work were also synthesized by Antarctic bacteria (i.e., styrene, dimethyl trisulfide, and dimethyl disulfide, as well as oxime-methoxy-phenyl). Moreover, this is also in agreement with the behaviour of *Paenibacilllus* sp. OVF10, which showed the weakest antibacterial potential, producing the lowest concentration of dimethyl disulfide (0.28%) and dimethyl trisulfide (0.03%) of all strains.

Oxime-, methoxy-phenyl- was present in all strains’ VOC profiles, in a concentration ranging from 0.04 to 8.29%. This compound was previously identified in a methanolic extract of *Pseudomonas aeruginosa* as a chemical with anti-bacterial activity [31].

All strains displayed a similar amount of monoterpenes emitted, in a range of concentration between 0.03 to 1.89%, with the exception of *Paenibacillus* sp. OVF10, for which monoterpenes were found in trace amounts (0.01%). The main components of the monoterpene fraction identified were α-pinene, 3-carene, p-cymene, and γ-terpinene. Numerous *in vitro* and *in vivo* research studies were conducted to evaluate the potential therapeutic uses of EOs extracted from different oregano species, detecting terpenes as the main components. In particular, EOs extracted from *O. vulgare* ssp. *vulgare*, which were found to be rich in γ-terpinene and p-cymene, exerted antioxidant, anti-inflammatory, antiproliferative, antitumor, and hypoglycemic activities [32].

Furthermore, 2-decenal was produced by all strains in a concentration ranging from 0.11 to 1.15%. This aldehyde is present in a wide variety of foods and it is considered to be a potent botanical nematicidal agent [33].

Among all the identified compounds, nine of them were pyrazines, which are known to have antifungal and nematicidal activities [34]. Only 2,5-dimethyl-pyrazine was produced by all strains, emitted in a range of concentrations between 0.13 and 15.30%. This substance was found to have significant antifungal activity against *Alternaria solani* and *Botrytis cinerea* [35]. The other eight pyrazine derivatives were detected only in *Paenibacillus* sp. OVF10, with the highest percentage for 2,3,4-trimethyl-5-propylpyrazine (83.92%). In agreement with the literature, *Paenibacillus* is a ubiquitously occurring bacterial genus with antagonistic activity against phytopathogens, which produces pyrazines such as 2-methyl-5-(1-methylethyl)-pyrazine, 2-(2 methylpropyl)-3-(1-methylethyl)pyrazine, and 2,3,4-trimethyl-5-propylpyrazine, as previously reported [36]. Previous studies revealed that *Paenibacillus*-emitted VOCs induced systemic resistance in *Arabidopsis* plants and enhanced plant growth at the same time [37]. Overall, alkyl-substituted pyrazines were detected as the main antimicrobial VOCs in the headspace of plant-associated *Paenibacillus polymyxa* isolates. This plant-associated isolate exhibited a high biocontrol potential due to its pronounced antagonistic activity against devastating plant pathogens [38].

Lasty, it was seen that only *Paenibacillus* sp. OVF10 produced 2-undecanone (0.13%). Previous SPME-GC/MS analysis conducted on *P. polymyxa* confirmed the presence of this ketone, detected with other VOCs emitted in order to study his nematicidal and fumigant activity against *Meloidogyne incognita* [39]. For further details on the biochemical mechanism of bacterial metabolites, refer to selected recent literature [40,41,42,43].

In conclusion, it can be hypothesized that all the endophytes considered in the present work might be involved in host plant protection against phytopathogens. This makes sense from a plant–bacterium symbiotic relationship point of view. In this context, among all the strains, OVF10 may be the most active one, inducing plant resistance and, potentially, also plant growth (acting as a plant growth promoter, PGP, bacterium), thanks to its production of different pyrazines [40,41]. Furthermore, the OVF10 ability to produce high levels of pyrazine could lead to other biotechnological applications considering the added value of such compounds for the food industry as flavour and fragrance [42].

Moreover, all the other seven endophytic strains (particularly the *Arthrobacter* sp. OVS8 strain) showed bactericidal and/or bacteriostatic activity against most of the Bcc strains and significantly higher production of dimethyl disulfide, dimethyl trisulfide, and monoterpenes compared to OVF10 [43]. Thus, it might be supposed that these seven endophytes could also have a role in the antibacterial properties of *O. vulgare* ssp. *vulgare*. Indeed, the same monoterpenes were also detected in the EOs extracted from the same *O. vulgare* plants from which endophytes were isolated [27], suggesting a role for *O. vulgare* ssp. *vulgare*-associated microbiome in the production of plant antimicrobial volatile compounds.

## 4. Materials and Methods

### 4.1. Bacterial Strains and Growth Conditions

The eight endophytic strains were isolated following the protocol reported in Castronovo et al. (2020). Bacteria were obtained from different compartments of *O. vulgare* ssp. *vulgare* plants (2 isolates from flower, 2 from leaf, and 4 from stem compartments). Isolates are referred to as OV followed by F, L, or S for flower, leaf, and stem districts, respectively. The selected isolates demonstrated the highest growth inhibitory activity against ten Bcc strains [27]. Six of them belong to the *Bacillus* genus (OVF22, OVL9, OVL24, OVS6, OVS21, and OVS26), while OVS8 and OVF10 belong to the *Arthrobacter* and *Paenibacillus* genera, respectively. Each strain was cultured on tryptic soy agar (TSA) medium (BioLife) for 48 h at 30 °C [27].

Ten strains of the *Burkholderia cepacia* complex (Bcc) collection, MDR bacteria able to resist different antibiotic classes, were selected based on their multiple drug resistance profile. The bacterial strains were isolated either from cystic fibrosis patients or the environment [20,44]. Each strain was cultured on LB agar medium (NaCl 10 g/L, yeast extract 5 g/L, tryptone 10 g/L, agar 15 g/L) for 48 h at 37 °C.

### 4.2. Analysis of the Antibacterial VOC Production through Cross-Streaking

Endophytes’ antibacterial activity was evaluated through cross-streaking. In this experiment, we used Petri dishes with a septum separating two compartments, to permit the growth of the tester and the target strains without any physical contact. Tester strains were streaked across one half of a TSA plate and grown at 30 °C for 48 h to allow the possible production of volatile antibacterial compounds. Target strains belonging to the Bcc were then all streaked perpendicularly to the tester strain and plates were incubated at 30 °C for a further 48 h. Additionally, target strains were streaked on half of a Petri plate in the absence of the tester and were allowed to grow at 30 °C for 48 h, as a growth control. The antagonistic effect was evaluated as the absence or reduction of the target strains growth compared to control plates. The different inhibition levels were indicated as follows: complete, strong, weak, and absence of inhibition [27].

### 4.3. Quantitative Analysis of VOC Antibacterial Activities

**Figure 4:**
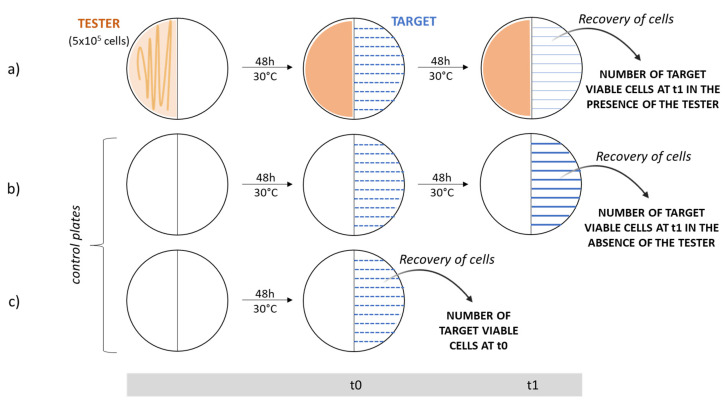
Schematic representation of the quantitative cross-streaking method. Target strain (blue) is streaked 10 times onto 3 half Petri dishes with septum. The cross-streaking plate (**a**) tests the ability of the tester strain (orange) to antagonize the growth of the target, while the growth control plate (**b**) permits one to verify its growth in the absence of the tester. In these last two cases, cells are recovered after incubation for 48 h at 30 °C (t_1_). The control plate (**c**) allows one to determine the number of cells streaked on the TSA medium; in this case, cells are immediately recovered for the viable count (t_0_).

The antibacterial activity of VOCs synthesized by each of the eight endophytes was evaluated through a quantitative cross-streaking method (Figure 4), by determining the number of viable cells of each target strain streaked on medium plates at the beginning of the experiment (t_0_, i.e., just after having streaked the target cells in the presence of the tester strain) and the number of viable cells at the end of the experiment (t_1_, i.e., after 48 h of incubation in the presence of the tester strains). The experiments were carried out as follows:

i. Single colonies from each endophyte were separately inoculated in 10 mL of fresh tryptic soy broth (TSB) medium (BioLife) and incubated at 30 °C overnight. The next morning, cells were counted in a Burker chamber and diluted in saline solution (0.9% w/v NaCl) in order to evenly spread about 5 × 10^5^ cells on one half of a TSA Petri plate with septum. A viable count of the cells plated was performed.
ii. Plates were incubated at 30 °C for 48 h to allow the production of volatile antibacterial organic compounds.
iii. Afterwards, a few colonies of each target strain (previously isolated and grown at 37 °C for 48 h on LB plates) were suspended in 100 µL of saline solution (0.9% *w*/*v* NaCl); then, a 1:100 dilution was prepared. The dilution was streaked 10 times perpendicularly to the septum on the opposite half of Petri dishes where the endophytes had grown.
iv. The same procedure was repeated on two TSA plates in the absence of the tester strains; these plates represented the control ones, which permitted us to determine the number of viable cells grown in the absence of the tester at the beginning (t_0_) and at the end of the experiment (t_1_).
v. All these plates were incubated at 30 °C for 2 days.
vi. To determine the number of cells just plated, the same dilutions were streaked 10 times on half of a Petri dish and cells were immediately recovered (t_0_) in 2 mL of saline solution with a spatula. The suspensions obtained were spread onto LB plates and incubated at 37 °C for 48 h to determine the viable titer.
vii. After 2 days, from cross-streaking plates and growth control plates, target strain cells were recovered in saline solution, as previously described. The suspensions obtained were properly diluted, spread onto LB plates, and incubated at 37 °C for 48 h for the viable count.

### 4.4. Identification of VOCs by Means of SPME-GC/MS

Each strain was cultured on tryptic soy agar (TSA) medium (BioLife, Sarasota, FL, USA) for 48 h at 30 °C [27]. Headspace vials were partially filled with the 2 g of solid medium containing colonies and sealed.

Bacterial volatile organic compounds (VOCs) produced by *O. vulgare* ssp. *vulgare* endophytic strains were extracted from the vial headspace and concentrated by solid-phase microextraction (SPME) before desorption in the GC injection port. Headspace extraction was performed with a 2.5 mL Syringe-HS (0.64-57-R-H, PTFE, GERSTEL) conditioned and held at 40 °C from sample collection to injection.

In the SPME, one Fibre Assembly was evaluated and used: 50/30 µm divinylbenzene (DVB)/ carbowax (CAR)/ polydimethylsiloxane (PDMS) (Supelco, Bellefonte, PA, USA). Fiber was exposed to bacterial culture in 20 mL SPME vial (75.5 × 22.5 mm) for 30 min at 40 °C, after 30 min of equilibration time. The desorption time was 5 min. Before use, fibre was conditioned and cleaned at 270 °C for 30 min, following instructions from Supelco^®^. Splitless injection was used.

Gas chromatographic analysis was performed using an Agilent 7000C GC (Agilent Technologies, Inc., Santa Clara, CA, USA) system equipped with a split/splitless injector, fitted with an Agilent HP5-MS UI capillary column (30 m × 250 μm; 0.25 μm film thickness) coupled to an Agilent triple quadrupole Mass Selective Detector MSD 5973 (Agilent Technologies, Inc., Santa Clara, CA, USA), with ionization voltage, 70 eV; electron multiplier energy, 2000 V; transfer line temperature, 270 °C. Solvent Delay: 0 min. Helium was used as the carrier gas (1 mL min^−1^). The oven program was as follows: temperature was initially kept at 40 °C for 5 min and then gradually increased to 250 °C at a rate of 2 °C/min, which was held for 15 min and finally raised to 270 °C at 10 °C/min. Samples were injected at 250 °C automatically. Interval scan: 35–450 *m*/*z*; Scan speed: 10,000 amu·s^−1^ (25 Hz).

The GC–MS mass spectrum data were analysed using MassHunter Qualitative Analysis B.06.00, and the database of National Institute Standard and Technology (NIST) was used to interpret analysed data. Comparison of the mass spectrum of the unidentified components released by the bacterial isolates was carried out against the mass spectrum of already-known components available in the NIST 11 MS library. The molecular weight and peak area percentage of unknown compounds were evaluated by the software as observed from the chromatogram.

## Figures and Tables

**Table 1:** Antibacterial activity of endophytic tester strains against target bacteria belonging to the Bcc. Values represent the mean percentage of number of viable cells of target strains in the presence of tester strains with respect to the number of viable cells grown in the absence of tester bacteria. The lower the value, the higher the antibacterial activity of the VOCs emitted by the endophyte.

	Mean (%)
*Arthrobacter* sp. OVS8	0.17
*Bacillus* sp. OVS26	4.14
*Bacillus* sp. OVF22	5.92
*Bacillus* sp. OVL9	19.68
*Bacillus* sp. OVS6	23.83
*Bacillus* sp. OVS21	30.96
*Bacillus* sp. OVL24	82.18
*Paenibacillus* sp. OVF10	1492.42

**Table 2:** Volatile organic compounds (VOCs) identified by SPME-GC/MS produced by *O. vulgare* ssp. *vulgare* endophytic strains. Results are expressed as mean relative abundance percentages (as obtained by dividing the area of each peak by the total area of the chromatogram peaks).

t_R_ (min)	Compounds	OVL9	OVL24	OVS6	OVS8	OVS21	OVS26	OVF10	OVF22
6.34	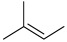 2-Butene, 2-methyl-	25.29	3.57	13.96	6.81	3.29	1.63	2.80	15.25
6.54	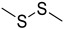 Dimethyl disulfide	20.28	60.51	35.14	84.55	68.41	8.41	0.28	43.00
7.95	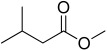 Butanoic acid, 3-methyl-, methyl ester	1.38	0.33	0.00	0.00	0.76	0.42	0.00	1.77
8.64	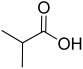 Propanoic acid, 2-methyl-	2.88	0.00	0.00	0.00	0.00	4.10	0.00	1.81
12.39	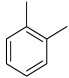 o-Xylene	0.40	0.36	1.56	0.07	0.24	0.35	0.01	0.30
12.99	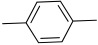 p-Xylene	0.74	0.59	3.93	0.13	0.37	0.78	0.02	0.59
13.44	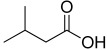 Butanoic acid, 3-methyl-	16.22	2.50	4.26	0.00	2.30	35.36	0.00	2.02
14.10	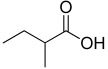 Butanoic acid, 2-methyl-	12.26	0.46	0.00	0.00	1.59	30.47	0.00	0.96
14.30	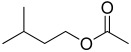 1-Butanol, 3-methyl-, acetate	1.07	0.59	6.31	0.13	0.26	1.60	0.01	0.49
14.40	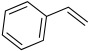 Styrene	2.54	2.75	3.46	0.57	12.99	2.81	0.05	2.48
16.08	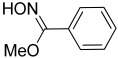 Oxime-, methoxy-phenyl-	3.36	3.12	8.29	0.47	0.55	3.13	0.04	6.35
16.11	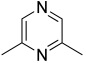 Pyrazine, 2,5-dimethyl-	8.12	7.19	3.36	1.33	6.29	5.15	0.08	15.30
17.35	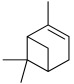 α-Pinene	0.27	0.17	0.46	0.04	0.03	0.12	0.01	0.19
17.99	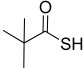 Propanethioic acid, 2,2-dimethyl-	0.61	13.32	1.10	1.33	0.55	0.47	0.01	1.22
19.84	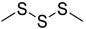 Dimethyl trisulfide	0.54	0.88	3.65	2.94	0.59	0.62	0.03	3.96
21.09	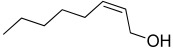 2-Octen-1-ol, (Z)-	0.64	0.82	0.16	0.04	0.26	0.27	0.39	0.74
22.99	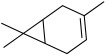 3-Carene	0.59	0.35	1.23	0.13	0.19	0.35	0.01	0.45
24.20	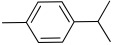 p-Cymene	0.23	0.22	1.02	0.07	0.12	0.21	0.01	0.24
24.58	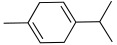 γ-Terpinene	0.46	0.38	1.89	0.10	0.17	0.29	0.01	0.31
24.74	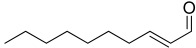 2-Decenal	0.77	0.85	1.15	0.12	0.54	0.94	0.11	0.79
25.74	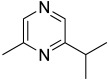 2-methyl-5-(1-methylethyl)-Pyrazine	0.00	0.00	0.00	0.00	0.00	0.00	7.92	0.00
31.40	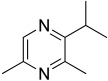 Pyrazine, 3,5-dimethyl-2-*i*-propyl-	0.00	0.00	0.00	0.00	0.00	0.00	0.04	0.00
32.29	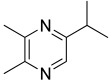 2,3-Dimethyl-5-*i*-propylpyrazine	0.00	0.00	0.00	0.00	0.00	0.00	0.03	0.00
32.64	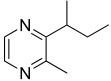 2-Isobutyl-3-methylpyrazine	0.00	0.00	0.00	0.00	0.00	0.00	0.01	0.00
34.11	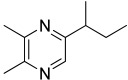 Pyrazine, 2,3-dimethyl-5-(1-methylpropyl)-	0.00	0.00	0.00	0.00	0.00	0.00	0.21	0.00
36.79	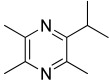 2,3,5-Trimethyl-6-propylpyrazine	0.00	0.00	0.00	0.00	0.00	0.00	83.92	0.00
37.34	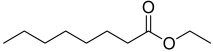 Octanoic acid, ethyl ester	0.86	0.70	4.74	0.67	0.34	1.59	0.06	1.22
40.89	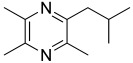 2-(2-Methylpropyl)-3,5,6-trimethylpyrazine	0.00	0.00	0.00	0.00	0.00	0.00	0.05	0.00
42.91	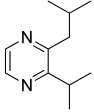 2-(2-Methylpropyl)-3-(1-methylethyl)pyrazine	0.00	0.00	0.00	0.00	0.00	0.00	3.56	0.00
44.04	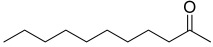 2-Undecanone	0.00	0.00	0.00	0.00	0.00	0.00	0.13	0.00
50.90	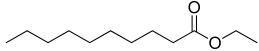 Decanoic acid, ethyl ester	0.48	0.34	4.34	0.36	0.17	0.93	0.02	0.56
61.73	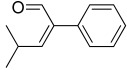 Benzeneacetaldehyde, α-(2-methylpropylidene)-	0.00	0.00	0.00	0.00	0.00	0.00	0.08	0.00
66.58	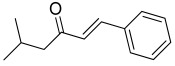 1-Hexen-3-one, 5-methyl-1-phenyl-	0.00	0.00	0.00	0.00	0.00	0.00	0.11	0.00

## Supplementary Materials

The following are available online at https://www.mdpi.com/article/10.3390/microorganisms10071424/s1. Table S1: Number of colony forming units of each target strain obtained at the beginning and at the end of the cross-streaking experiments in the absence or in the presence of the endophytic strains.
