# Supplementary material for: Endophytic Bacteria and Essential Oil from Origanum vulgare ssp. vulgare Share Some VOCs with an Antibacterial Activity
Source: Microorganisms. 2022 Jul 14;10(7):1424. doi: 10.3390/microorganisms10071424 (PMC9320186; doi:10.3390/microorganisms10071424)
Supplement: Supplementary file 1 [file microorganisms-10-01424-s001.zip › microorganisms-1788634-supplementary.pdf]

# Supplementary Material

# Endophytic bacteria and essential oil from Origanum vulgare ssp. vulgare share some VOCs with an antibacterial activity

**Giulia Polito[1†], Giulia Semenzato[2†], Sara Del Duca[2], Lara Mitia Castronovo[2], Alberto Vassallo[2], Sofia Chioccioli[2], Duccio Borsetti[2], Vittoria Calabretta[2], Anna Maria Puglia[1], Renato Fani[2] and Antonio Palumbo Piccionello[1,*]**

[1]Department of Biological, Chemical and Pharmaceutical Sciences and Technologies-STEBICEF, University of Palermo, Viale delle Scienze Ed.17, 90128, Palermo, Italy; giulia.polito@unipa.it, a.maria.puglia@unipa.it
[2]Department of Biology, University of Florence, Via Madonna del Piano 6, Sesto Fiorentino, 50019 Florence, Italy; giulia.semenzato@unifi.it, sara.delduca@unifi.it, l.castronovo@student.unisi.it; alberto.vassallo@unicam.it; sofia.chioccioli@unifi.it; duccio.borsetti@stud.unifi.it; vittoria.calabretta@stud.unifi.it; renato.fani@unifi.it
*Correspondence: antonio.palumbopiccionello@unipa.it; Tel.: +3909123897544
†Authors equally contributed to this work.

**Table S1.** Number of colony forming units (CFU) of each target strain obtained at the beginning ($t_0$) and at the end ($t_1$) of the cross-streaking experiments in the absence (C+) or in the presence (*vs* TESTER) of the endophytic strains.

| | $t_0$ | $t_1$ *vs* **OVS8** | C+ | $t_1$ compared to C+ (%) |
|---|---|---|---|---|
| FCF3 | 5.9 x $10^3$ | 7.2 x $10^4$ | 5.0 x $10^8$ | 0.0144 |
| FCF23 | 1.6 x $10^3$ | <$10^2$ | 9.0 x $10^5$ | 0.0110 |
| LMG13010 | 3.0 x $10^3$ | 4.3 x $10^5$ | 2.7 x $10^8$ | 0.1593 |
| LMG16656 | 8.5 x $10^3$ | <$10^2$ | 4.8 x $10^7$ | 0.0002 |
| LMG21462 | 2.0 x $10^3$ | <$10^2$ | 1.1 x $10^8$ | 0.0001 |
| LMG24506 | 1.3 x $10^4$ | <$10^2$ | 1.1 x $10^8$ | 0.0001 |
| LMG1222 | 2.9 x $10^3$ | 5.7 x $10^4$ | 7.5 x $10^6$ | 0.7600 |
| LMG17588 | 3.0 x $10^3$ | 6.3 x $10^2$ | 6.6 x $10^6$ | 0.0095 |
| LMG19182 | 7.6 x $10^3$ | <$10^2$ | 8.0 x $10^5$ | 0.0124 |
| LMG19230 | 2.7 x $10^3$ | 5.1 x $10^5$ | 6.7 x $10^7$ | 0.7612 |
| | | | **Mean** | **0.1728** |
| | $t_0$ | $t_1$ *vs* **OVF10** | C+ | $t_1$ compared to C+ (%) |
| FCF3 | 5.9 x $10^3$ | 2.0 x $10^8$ | 5.0 x $10^8$ | 40.0000 |
| FCF23 | 1.6 x $10^3$ | 5.6 x $10^7$ | 9.0 x $10^5$ | 6222.2222 |
| LMG13010 | 3.0 x $10^3$ | 1.4 x $10^8$ | 2.7 x $10^8$ | 51.8519 |
| LMG16656 | 8.5 x $10^3$ | 2.7 x $10^6$ | 4.8 x $10^7$ | 5.6250 |
| LMG21462 | 2.0 x $10^3$ | 3.9 x $10^5$ | 1.1 x $10^8$ | 0.3545 |
| LMG24506 | 1.3 x $10^4$ | 1.7 x $10^9$ | 1.1 x $10^8$ | 1545.4545 |
| LMG1222 | 2.9 x $10^3$ | 3.8 x $10^8$ | 7.5 x $10^6$ | 5066.6667 |
| LMG17588 | 3.0 x $10^3$ | 1.1 x $10^8$ | 6.6 x $10^6$ | 1666.6667 |
| LMG19182 | 7.6 x $10^3$ | 2.4 x $10^6$ | 8.0 x $10^5$ | 300.0000 |
| LMG19230 | 2.7 x $10^3$ | 1.7 x $10^7$ | 6.7 x $10^7$ | 25.3731 |

| | | | | |
|---|---|---|---|---|
| | | | **Mean** | **1492.4215** |
| | $t_0$ | $t_1$ *vs* **OVS6** | C+ | $t_1$ compared to C+ (%) |
| FCF3 | 5.9 x $10^3$ | 2.8 x $10^5$ | 5.0 x $10^8$ | 0.0560 |
| FCF23 | 1.6 x $10^3$ | 1.5 x $10^4$ | 9.0 x $10^5$ | 1.6667 |
| LMG13010 | 3.0 x $10^3$ | 1.1 x $10^8$ | 2.7 x $10^8$ | 40.7407 |
| LMG16656 | 8.5 x $10^3$ | 1.8 x $10^5$ | 4.8 x $10^7$ | 0.3750 |
| LMG21462 | 2.0 x $10^3$ | 3.3 x $10^2$ | 1.1 x $10^8$ | 0.0003 |
| LMG24506 | 1.3 x $10^4$ | 8.8 x $10^4$ | 1.1 x $10^8$ | 0.0800 |
| LMG1222 | 2.9 x $10^3$ | 1.1 x $10^6$ | 7.5 x $10^6$ | 14.6667 |
| LMG17588 | 3.0 x $10^3$ | 6.7 x $10^6$ | 6.6 x $10^6$ | 101.5152 |
| LMG19182 | 7.6 x $10^3$ | 8.4 x $10^2$ | 8.0 x $10^5$ | 0.1050 |
| LMG19230 | 2.7 x $10^3$ | 5.3 x $10^7$ | 6.7 x $10^7$ | 79.1045 |
| | | | **Mean** | **23.8310** |
| | $t_0$ | $t_1$ *vs* **OVL24** | C+ | $t_1$ compared to C+ (%) |
| FCF3 | 5.9 x $10^3$ | 8.7 x $10^5$ | 5.0 x $10^8$ | 0.1740 |
| FCF23 | 1.6 x $10^3$ | 3.8 x $10^5$ | 9.0 x $10^5$ | 42.2222 |
| LMG13010 | 3.0 x $10^3$ | 2.4 x $10^7$ | 2.7 x $10^8$ | 8.8889 |
| LMG16656 | 8.5 x $10^3$ | N.D. | 4.8 x $10^7$ | N.D. |
| LMG21462 | 2.0 x $10^3$ | 7.0 x $10^2$ | 1.1 x $10^8$ | 0.0006 |
| LMG24506 | 1.3 x $10^4$ | 1.1 x $10^6$ | 1.1 x $10^8$ | 1.0000 |
| LMG1222 | 2.9 x $10^3$ | 2.0 x $10^6$ | 7.5 x $10^6$ | 26.6667 |
| LMG17588 | 3.0 x $10^3$ | 3.3 x $10^7$ | 6.6 x $10^6$ | 500.0000 |
| LMG19182 | 7.6 x $10^3$ | 9.2 x $10^4$ | 8.0 x $10^5$ | 11.5000 |
| LMG19230 | 2.7 x $10^3$ | 1.0 x $10^8$ | 6.7 x $10^7$ | 149.2537 |
| | | | **Mean** | **82.1896** |
| | $t_0$ | $t_1$ *vs* **OVL9** | C+ | $t_1$ compared to C+ (%) |
| FCF3 | 2.8 x $10^3$ | 1.5 x $10^4$ | 7.7 x $10^7$ | 0.0195 |
| FCF23 | 2.3 x $10^2$ | 9.2 x $10^4$ | 4.2 x $10^6$ | 2.1905 |
| LMG13010 | 1.9 x $10^3$ | 3.5 x $10^6$ | 1.8 x $10^8$ | 1.9444 |
| LMG16656 | 3.5 x $10^2$ | $<10^3$ | 8.2 x $10^7$ | 0.0012 |
| LMG21462 | 1.9 x $10^3$ | $<10^3$ | 6.4 x $10^7$ | 0.0016 |
| LMG24506 | 7.7 x $10^3$ | 1.4 x $10^3$ | 1.4 x $10^8$ | 0.0010 |
| LMG1222 | 7.9 x $10^2$ | $<10^5$ | 7.4 x $10^7$ | 0.1351 |
| LMG17588 | 7.6 x $10^2$ | 2.2 x $10^5$ | 1.3 x $10^7$ | 1.6923 |
| LMG19182 | 1.6 x $10^3$ | 1.1 x $10^6$ | $<10^6$ | 110.0001 |
| LMG19230 | 4.0 x $10^2$ | 2.1 x $10^7$ | 2.6 x $10^7$ | 80.7692 |
| | | | **Mean** | **19.6755** |
| | $t_0$ | $t_1$ *vs* **OVS21** | C+ | $t_1$ compared to C+ (%) |
| FCF3 | 2.8 x $10^3$ | N.D. | 7.7 x $10^7$ | N.D. |
| FCF23 | 2.3 x $10^2$ | 6.8 x $10^4$ | 4.2 x $10^6$ | 1.6190 |
| LMG13010 | 1.9 x $10^3$ | 5.2 x $10^6$ | 1.8 x $10^8$ | 2.8889 |
| LMG16656 | 3.5 x $10^2$ | 2.1 x $10^5$ | 8.2 x $10^7$ | 0.2561 |
| LMG21462 | 1.9 x $10^3$ | $<10^3$ | 6.4 x $10^7$ | 0.0016 |
| LMG24506 | 7.7 x $10^3$ | 5.6 x $10^3$ | 1.4 x $10^8$ | 0.0040 |
| LMG1222 | 7.9 x $10^2$ | 3.9 x $10^7$ | 7.4 x $10^7$ | 52.7027 |
| LMG17588 | 7.6 x $10^2$ | 1.2 x $10^7$ | 1.3 x $10^7$ | 92.3077 |
| LMG19182 | 1.6 x $10^3$ | 5.2 x $10^5$ | $<10^6$ | 52.0001 |
| LMG19230 | 4.0 x $10^2$ | 2.0 x $10^7$ | 2.6 x $10^7$ | 76.9231 |
| | | | **Mean** | **30.9670** |
| | $t_0$ | $t_1$ *vs* **OVF22** | C+ | $t_1$ compared to C+ (%) |
| FCF3 | 2.8 x $10^3$ | 7.5 x $10^4$ | 7.7 x $10^7$ | 0.0974 |
| FCF23 | 2.3 x $10^2$ | 4.8 x $10^3$ | 4.2 x $10^6$ | 0.1143 |

| | | | | |
|---|---|---|---|---|
| LMG13010 | $1.9 \times 10^{3}$ | $2.9 \times 10^{5}$ | $1.8 \times 10^{8}$ | 0.1611 |
| LMG16656 | $3.5 \times 10^{2}$ | $<10^{3}$ | $8.2 \times 10^{7}$ | 0.0012 |
| LMG21462 | $1.9 \times 10^{3}$ | $<10^{3}$ | $6.4 \times 10^{7}$ | 0.0016 |
| LMG24506 | $7.7 \times 10^{3}$ | $<10^{3}$ | $1.4 \times 10^{8}$ | 0.0007 |
| LMG1222 | $7.9 \times 10^{2}$ | $2.4 \times 10^{7}$ | $7.4 \times 10^{7}$ | 32.4324 |
| LMG17588 | $7.6 \times 10^{2}$ | $3.6 \times 10^{4}$ | $1.3 \times 10^{7}$ | 0.2769 |
| LMG19182 | $1.6 \times 10^{3}$ | $2.0 \times 10^{5}$ | $<10^{6}$ | 20.0000 |
| LMG19230 | $4.0 \times 10^{2}$ | $1.6 \times 10^{6}$ | $2.6 \times 10^{7}$ | 6.1538 |
| | | | **Mean** | **5.9235** |
| | $t_0$ | $t_1$ *vs* **OVS26** | C+ | $t_1$ compared to C+ (%) |
| FCF3 | $2.8 \times 10^{3}$ | $1.3 \times 10^{4}$ | $7.7 \times 10^{7}$ | 0.0169 |
| FCF23 | $2.3 \times 10^{2}$ | $1.4 \times 10^{3}$ | $4.2 \times 10^{6}$ | 0.0333 |
| LMG13010 | $1.9 \times 10^{3}$ | $6.6 \times 10^{5}$ | $1.8 \times 10^{8}$ | 0.3667 |
| LMG16656 | $3.5 \times 10^{2}$ | $1.3 \times 10^{3}$ | $8.2 \times 10^{7}$ | 0.0016 |
| LMG21462 | $1.9 \times 10^{3}$ | $<10^{3}$ | $6.4 \times 10^{7}$ | 0.0016 |
| LMG24506 | $7.7 \times 10^{3}$ | $<10^{3}$ | $1.4 \times 10^{8}$ | 0.0007 |
| LMG1222 | $7.9 \times 10^{2}$ | $1.4 \times 10^{4}$ | $7.4 \times 10^{7}$ | 0.0189 |
| LMG17588 | $7.6 \times 10^{2}$ | $3.0 \times 10^{3}$ | $1.3 \times 10^{7}$ | 0.0231 |
| LMG19182 | $1.6 \times 10^{3}$ | $1.4 \times 10^{5}$ | $<10^{6}$ | 14.0000 |
| LMG19230 | $4.0 \times 10^{2}$ | $7.0 \times 10^{6}$ | $2.6 \times 10^{7}$ | 26.9231 |
| | | | **Mean** | **4.1386** |
